# Routes and methods of neural stem cells injection in cerebral ischemia

**DOI:** 10.1002/ibra.12122

**Published:** 2023-08-06

**Authors:** Xing‐Yu Yang, Xiao Zhang, Jun‐Feng Cao, Mei Wu, Sheng‐Yan Chen, Li Chen

**Affiliations:** ^1^ School of Clinic Medicine Chengdu Medical College Chengdu Sichuan China; ^2^ School of Basic Medicine Chengdu Medical College Chengdu Sichuan China; ^3^ Institute of Neurological Disease, Translational Neuroscience Center, West China Hospital Sichuan University Chengdu Sichuan China

**Keywords:** cerebral ischemia, injection doses, injection routes, neural stem cells

## Abstract

Cerebral ischemia is a serious cerebrovascular disease with the characteristics of high morbidity, disability, and mortality. Currently, stem cell therapy has been extensively applied to a wide range of diseases, including neurological disorders, autoimmune deficits, and other diseases. Transplantation therapy with neural stem cells (NSCs) is a very promising treatment method, which not only has anti‐inflammatory, antiapoptotic, promoting angiogenesis, and neurogenesis effects, but also can improve some side effects related to thrombolytic therapy. NSCs treatment could exert protective effects in alleviating cerebral ischemia‐induced brain damage and neurological dysfunctions. However, the different injection routes and doses of NSCs determine diverse therapeutic efficacy. This review mainly summarizes the various injection methods and injection effects of NSCs in cerebral ischemia, as well as proposes the existing problems and prospects of NSCs transplantation.

## Graphical abstract

Cerebral ischemia is a serious cerebrovascular disease with the characteristics of high morbidity, disability, and mortality. Neural stem cells (NSCs) transplantation therapy is a very promising treatment method, which not only has anti‐inflammatory, antiapoptotic, promoting angiogenesis, and neurogenesis effects, but also can improve some side effects related to thrombolytic therapy. At the same time, NSCs treatment could exert protective effects in alleviating cerebral ischemia‐induced brain damage and neurological dysfunctions. However, the different injection routes and doses of NSCs determine diverse therapeutic efficacy. We summarized the injection routes of NSCs, which mainly include intravenous, arterial, as well as intracerebral injection via ventricles, striatum, corpus callosum, cerebral cortex, hippocampus, and infarct area. Moreover, the injection methods and doses of transplanted NSCs in cerebral ischemia were also introduced.

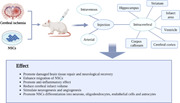

## 1 INTRODUCTION

Hypoxic–ischemic (HI) brain injury is a serious cause of morbidity and mortality in adults and newborns, including neonatal HI brain damage and adult ischemic stroke.^1^ Ischemic stroke, also known as cerebral ischemia, is an important type of stroke. Moreover, it is a severe cerebrovascular disease with high mortality and morbidity.^2^ In industrialized countries, stroke is the third leading cause of death. Every year, more than 795,000 people in the USA have a stroke. About 87% of all strokes are ischemic strokes, an acute arterial blood flow disorder in the brain.^3^ Histopathological changes of cerebral ischemia include cortical atrophy, cortical and hippocampal neuronal degeneration, leukocytosis, glial cell proliferation, capillary bed changes, and so forth. Moreover, after cerebral ischemia, the infarct volume is larger, the functional recovery is limited, as well as the angiogenesis and neurogenesis are reduced.^4^ Therefore, for most stroke patients, neuron death is inevitable, and how to actively and effectively promote the recovery of neurological function is still the focus of current stroke research.^5^, ^6^ At present, the main treatments include ultra‐early thrombolysis, acute neuron protection therapy, intravenous therapy, antiplatelet drugs, anticoagulation therapy, and so forth. However, the narrow time window limits these treatment measures.^5^ Hemorrhagic infarction and cerebral hematoma are terrible events that may occur after cerebral ischemia. The thrombolytic agent may be an effective stroke therapy, but it may also promote bleeding complications after ischemic stroke.^7^

Stem cells can play an important role in neuroprotection, angiogenesis, inflammation, and immune response to a certain extent.^8^ Moreover, stem cell transplantation is effective in a wide range of disorders, including neurological diseases, autoimmune diseases, and other diseases. It is the safest, nontoxic treatment method with the least side effects. It is generally believed that neural stem cells (NSCs) may be an ideal choice for establishing a stem cell bank to continuously supply the required neurons, astrocytes, or oligodendrocytes.^9^ Transplanted NSCs exert beneficial effects not only via structural replacement but also via immunomodulatory and/or neurotrophic actions.^10^ Meanwhile, NSC transplantation attenuates apoptosis and improves neurological functions after cerebral ischemia.^11^ Since the NSCs can be engrafted through various routes with different doses, this review mainly summarizes the efficacy of NSCs transplantation with different injection routes and transplantation methods.

## 2 OVERVIEW OF NSCs

### 2.1 Sources and characteristics of NSCs

NSCs refer to cells that are self‐renewing as well as have the potential to differentiate into neurons, astrocytes, oligodendrocytes, and other multidirectional differentiation potentials. Furthermore, these show up a very low risk of malignant transformation.^12^ NSCs can be directly derived from nervous tissues: from the cerebral cortex, hippocampus, striatum, subependymal area, subventricular area, spinal cord of embryonic mammals, and other parts of adult animals, NSCs have successfully isolated and cultured. Besides, NSCs can be derived from the differentiation of embryonic stem cells, bone marrow mesenchymal stem cells, cord blood mesenchymal stem cells, adipose mesenchymal stem cells, embryonic olfactory ensheathing cells, and induced pluripotent stem cells.^13^ NSCs have abilities to self‐maintain and proliferate and possess a variety of cell differentiation potentials.^14^

### 2.2 Necessity of NSCs transplantation in cerebral ischemia

Endogenous NSCs in some areas of the adult central nervous system are mainly distributed in the hippocampus and subventricular zone (SVZ) of young adults. However, these endogenous NSCs are in a dormant state. Cerebral ischemia stimulates cell proliferation and neuroblast formation in the hippocampus and subependymal zone, thereby boosting that endogenous NSCs migrate to the ischemic area and differentiate into neurons to integrate into the damaged area to play a role.^15^ After cerebral ischemia, increased proliferation and differentiation of NSCs were observed in the hippocampus and subependymal zone. However, stimulation of endogenous NSCs in adults has a limited effect on the recovery of neurological function in patients with ischemic stroke.^16^ Investigators also use various in vitro culture methods to obtain a certain number of exogenous NSCs, and most of the exogenous NSCs are derived from embryos or fetuses. When human NSCs are implanted into the brain of rats with cerebral ischemia, these cells can migrate to the ischemic area, proliferate, and differentiate into neurons, as well as improve sensorimotor function,^17^ which can significantly repair ischemic brain injury. However, NSCs transplantation into the human body is still in the primary stage of experimental research. Although some clinical results have been achieved, the technology is not mature enough. Large‐sample, multi‐center, randomized controlled clinical trials and large‐scale clinical trials for validation are still lacking.

## 3 CURRENT RESEARCH PROGRESS OF NSCs INJECTION IN CEREBRAL ISCHEMIA

**Figure 1:**
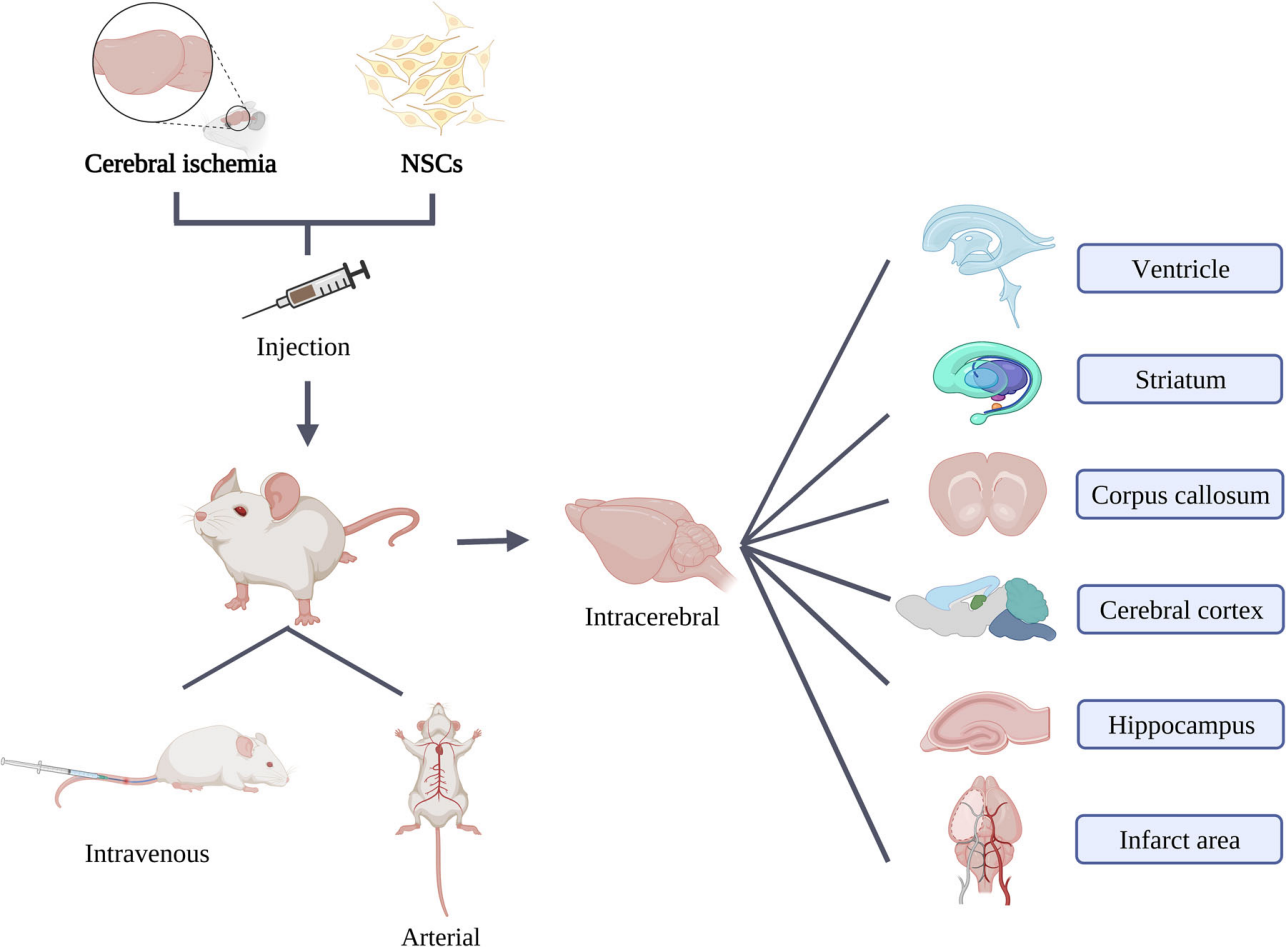
Routes of neural stem cells (NSCs) injection in cerebral ischemia. [Color figure can be viewed at wileyonlinelibrary.com]

We summarized the injection routes of NSCs, which mainly include intravenous, arterial, as well as intracerebral injection via ventricles, striatum, corpus callosum, cerebral cortex, hippocampus, and infarct area (Figure 1). Below we mainly introduce the routes, methods, and doses of transplanted NSCs in cerebral ischemia.

### 3.1 Intravenous

*Tail vein*: 1 × 10^3^ NSCs were administered using a 1 mL syringe along the caudal vein^18^ or approximately 5 × 10^6^ NSCs in 200 μL phosphate‐buffered saline (PBS) or an equal volume of PBS into the rat tail vein of adult male SD rats^19^; *Jugular vein*: Rats were injected with 1 mL of NSC suspension (1 × 10^5^ cells) through the jugular vein in Wistar rats^20^ (Table 1).

**Table 1:** Representative experimental studies of intravenous and arterial NSC transplantation in cerebral ischemia.

Route	Timing of transplantation	Cell type	Dose	Species	Model/Duration	Major finding	Reference
Intravenous	24 h	Mouse C17.2 NSCs (from the cerebellum)	5 × 10^6^	SD rat	tMCAO/120 min	Promoting the migration, proliferation, and differentiation of NSCs in the brain into neurons and astrocytes, improving functional recovery	[19]
	7 days	Mouse NSCs (from the fetal brain)	1 × 10^5^	Wistar rat	Hypoxia–ischemia stroke (8%O_2_)/3.0 h	Significantly improving pathological damage, restoring neurobehavioral function	[21]
	7 days	Mouse NSCs (from the embryonic hippocampus)	1 × 10^3^	SD rat	Hypoxia–ischemia stroke (8%O_2_)/2.5 h	Acupuncture + NSC combined therapy can significantly improve the motor learning and memory abilities of young rats with cerebral palsy and reduce nerve cell apoptosis	[18]
Arterial	24 h	Mouse NSCs (from the embryo)	2 × 10^6^	SD rat	MCAO	Migrating to the ischemic area, differentiating into astrocytes and neurons, improving neurological function after cerebral ischemia	[22]
	48 h	Mouse C17.2 NSCs (from the cerebellum)	3 × 10^5^	C57/BL6 Mouse	Hypoxia–ischemia stroke (8%O_2_)/20 min	Increasing their homing to the ischemic brain tissue, significantly improving sensorimotor recovery	[17]
	3 days	Mouse NSCs (from the subventricular zone (SVZ)）	1 × 10^6^	C57BL/6 Mouse	tMCAO/60 min	Differentiating into glial or neuron, mainly migrating to the ipsilateral hemisphere	[23]
Intracerebral	3 days	Mouse NSCs (from the cortex)	1 × 10^6^	SD rat	ICH/2 min	Significantly better performance in the mouse limb placement test at 3 weeks and later	[24]

Abbreviations: IA, intra‐arterial; ICH, intracerebral hemorrahge; IV, intravenous; MCAO, middle cerebral artery occlusion; NSCs, neural stem cells; SD, Sprague Dawley; tMCAO, transient middle cerebral artery occlusion.

### 3.2 Arterial

*Common carotid artery*: 3 × 10^5^ NSCs were injected with a 10 μL Hamilton syringe and 33 g needle in C57BL/6 mice^17^. *Left common carotid artery*: 1 × 10^6^ green fluorescent protein (GFP)‐NSCs were injected in 10–12‐week‐old C57BL/6 mice through the catheter with a syringe pump at a rate of 20 μL/min for 5 min and then rinsed with 50–100 μL PBS at the same rate. *External carotid artery*: 5 × 10^6^ NSCs in 100 µL of PBS at 50 µL/min for 2 min and then rinsed with 50–100 µL of PBS at the same speed^23^; *Internal carotid artery*: 2 × 10^6^ cells were injected in adult male SD rats^22^ (Table 1).

### 3.3 Intracerebral

Using the anterior bregma of the skull as the reference point, the transplantation point of 12‐week‐old mice is set 2 mm to the right of the sagittal suture and 2 mm behind the coronal suture. After drilling the implantation site to a thickness of 50 μm, 25 μL of NSCs (1 × 10^4^ cells/μL) was injected at the implantation site with an invasion depth of 3 mm. The injection was completed within 5 min, and the needle was kept at the injection site for another 5 min.^25^ The needle was inserted into the bone hole of 14‐day‐old SD rats at 5 mm below the surface of the skull and then 5 μL of cell suspension was injected at the final cell concentration of 2 × 10^7^ cells/100 μL. The needle was drawn out slowly after 10 min.^24^ A Hamilton syringe connected to an UltraMicroPump3 (UMP3) with an SYS‐Micro4 controller was used to inject a total volume of 3 μL of 3 × 10^5^ cell suspension at a rate of 1 μL/min in P7 SD rats. Then, the needle was kept in the skull for another 2 min.^26^ A burr hole (1 mm) was made on the right side of the cranium in adult male SD rats at a rate of 5 μL/min (0.5 mm posterior and 3.5 mm to the right of the bregma; 4.5 mm below the dura).^27^ Positioned 500 μm from the dural surface from the bregma, a burr hole was made using a small dental drill, and a suspension of 1 × 10^4^ cells/μL was injected stereotactically at a depth of 500 μm from the dural surface at 3 mm lateral and 1.0 mm dorsal from the bregma in 6‐week‐old mice^28^ (Tables 1 and 2).

**Table 2:** Representative experimental studies of intracerebral (ventricular and striatum) NSCs transplantation in cerebral ischemia.

Route	Timing of transplantation	Cell type	Dose	Species	Model/Duration	Major finding	Reference
Intracerebral	7 days	Mouse adult neural stem/progenitor cells (from cortex‐derived)	1 × 10^4^	CB‐17/Icr‐^+/+^Jcl Mouse (CB‐17 Mouse) and CB‐17/Icr‐scid/scid Jcl Mouse (SCID Mouse)	dMCAO	The transplantation of endothelial cells and cortex‐derived, injury‐induced adult neural stem/progenitor cells promotes survival, proliferation, and neuronal differentiation in the ischemic brain and improves the recovery of cortical function	[28]
Intracerebral							
Ventricular	7 days	Mouse NSCs (from the hippocampus)	1 × 10^6^	C57BL/6 Mouse	tMCAO/60 min	The transplanted NSCs migrate to the diseased tissue through the corpus callosum. The silence of circHIPK2 in it promotes neuronal differentiation and enhances functional recovery after stroke	[29]
Striatum	30 min	Mouse NSCs (from the hippocampus)	2 × 10^5^	SD rat	tMCAO/90 min	Promoting the differentiation of endogenous NSCs in the subventricular zone of the lateral ventricle toward neurons, reducing neuronal apoptosis, and improving the damage of ischemia to the neurological function of rats	[30]
	24 h	Mouse NSCs (from the telomere)	1 × 10^6^	SD rat	tMCAO/2 h	The transplanted NSCs can not only enhance the endogenous VEGF secretion of astrocytes and ECs but also protect endogenous neurons from apoptosis, promote angiogenesis and neurogenesis, thereby reducing infarct volume and improving neurobehavioral recovery	[31]

Abbreviation: NSCs, neural stem cells.

#### 3.3.1 Ventricular

NSCs (1 × 10^6^) with a total volume of 2 μL were injected into the left ventricle of adult male C57BL/6J mice at a rate of 0.1 L/min at these coordinates: front and rear, −0.3 mm; transverse, +1.0 mm; and ventral, +2.2 mm.^29^ NSCs were injected into the left side (injury side) of the cerebral ventricle in SD rats, and the injection site was different according to the weight of the young rat as follows: anteroposterior (AP), −1 mm; medial lateral (ML), −1.0 to −1.5 mm; and dorsal ventral (DV), −3.5 to −4.0 mm. Five microliters (cell concentration of 1 × 10^5^ cells/μL) was injected via a microsyringe, slowly injected at a uniform speed, the needle was stopped for 5 min before withdrawing the needle, and then was withdrawn slowly^32^ (Table 2).

#### 3.3.2 Striatum

Two milliliters of NSCs (2 × 10^5^) using a NO.26 Hamilton syringe at the flow rate of 0.5 μL/min was transplanted into the right striatum of 14‐day‐old SD rats. The coordinates from the anterior fontanelle are as follows: AP, −1.0 mm; ML, +3.0 mm; and DV, −5.0 mm. The needle was left for 5 min after each injection and then slowly removed at a rate of 1 mm/min.^33^ NSCs (1 × 10^6^ in 25 μL of PBS) were injected stereotaxically into the striatum of the ipsilateral hemisphere in 3‐ and 24‐month‐old male SD rats with the coordinates as follows: AP, −0.5 mm; lateral (L), −3.0 mm; and vertical (V), −5.0 mm.^31^ NSCs (1 × 10^6^ cells in 30 μL of PBS) were injected into the striatum of adult male SD rats, which was 0.8 mm behind the anterior fontanelle, 3.4 mm to the left of the sagittal suture, and the injection depth was 5.8 mm. The injection was given immediately after reperfusion, the time was controlled within 10 min, a constant speed was maintained, and the needle was pulled out after holding the needle for another 5 min.^34^ Stereotactic transplantation of 1–2 μL of NSCs at a concentration of 5 × 10^4^ cells/μL (low dose) or 1 × 10^4^ cells/μL (high dose) was performed. These transplantations were carried out at one site with a 5 × 10^5^ cell dose or at two sites with 5 × 10^5^ and 1 million cell doses, within the lesioned striatum in adult male SD rats at the following coordinates: AP, +0.5 mm; ML, +3.0 mm; DV, −5.0; AP, −0.5 mm; ML, +3.0 mm; and DV, −5.0.^35^ 2 × 10^4^ cells/μL of neurosphere cells at the rate of 1 μL/min was transplanted at two points in the expected area of striatal infarction of adult male Wistar rats: AP, +0.8; L, −4.0; and V, −5.6 mm and AP, +1.5; L, −3.0; and V, −5.0 mm below the dura.^36^ The brain stereotaxic instrument was selected to locate the left striatum of female SD rats, and the coordinate zero point was the Bregma point: AP, 0 mm; ML, 2.0 mm; and DV, 4.5 mm. Ten microliters of NSCs were injected into a microsyringe at 0.15 μL/s, and the cell concentration was 2 × 10^10^ L^−1^. After the injection, the needle was retained for 20 min.^37^ A total of 3 × 10^5^ NSCs dissolved in 10 μL of PBS was injected into the left striatum (AP, 0 mm; ML, −2.5 mm; DV, +3 mm relative to the bregma) of adult male mice, using a micropump. Then, the needle in the brain was kept for another 5 min.^38^ The head was fixed on the brain stereotaxic instrument, the former fontanelle was the coordinate zero point, and the left striatum of adult male SD rats was positioned (AP, 0 mm; ML, +3.14 mm; DV, +5.4 mm). NSCs were injected at a rate of 1 μL/min using a microsyringe delivering a total of 10 μL with a cell concentration of about 1 × 10^6^cells/L. The injection was stopped and the needle was retained for 10 min.^39^ Cells (5 × 10^5^ cells, 5 μL) were transplanted into the right striatum of neonatal Wistar rats (AP, 0.0 mm; ML, +2.0 mm; DV, +3.5 mm).^40^ The coordinates of the striatal graft site were 0.5 mm anterior to the bregma, 1.8 mm to the right of the suture of the skull, and 3.5 mm below the plane of the skull. 5 × 10^5^ (6 μL) NSCs were implanted from here in 8–10‐week‐old mice^41^ (Tables 2 and 3).

**Table 3:** Representative experimental studies of intracerebral (striatum) NSC transplantation in cerebral ischemia.

Route	Timing of transplantation	Cell type	Dose	Species	Model/Duration	Major finding	Reference
Intracerebral							
Striatum	48 h	Mouse NSCs (from the cortex and ganglion uplift)	2 × 10^5^	SD rat	tMCAO/2 h	The combination of PFT‐α and NSC transplantation can increase the survival rate of NSCs after transplantation in rat stroke models and promote the recovery of nerve function in MCAO rats	[33]
	72 h	Mouse NSCs (from the brain tissue)	10	SD rat	tMCAO/2 h	DAPT can promote the differentiation of transplanted NSCs into neurons and has a neuroprotective effect on NSC transplantation in rats with cerebral ischemia	[39]
	4 days	Mouse NSCs (from the cortex)	3 × 10^5^	ICR Mouse	tMCAO/60 min	Improving neurological recovery	[38]
	7 days	Human NSCs (from embryonic brain tissue)	5 × 10^5^	C57B6/L Mouse	tMCAO/60 min	Improving the recovery of neurological function in mice after ischemia and reducing infarct volume	[41]
	7 days	H7 human embryonic stem cell lines (WiCell)	5 × 10^4^	SD rat	tMCAO/1.5 h	Promoting the formation of oligodendrocytes and astrocytes	[35]
	10 days	Human iPSCs (iPS‐S‐01) and human ESCs (HuES17)	1 × 10^6^	SD rat	tMCAO/2 h	NSCs derived from iPSC differentiate into neurons and astrocytes, migrate to the ischemic area, and improve the behavior and sensorimotor function of rats	[34]
	1 week	Mouse NSCs (from the olfactory bulb)	3 × 10^5^	SD rat	tMCAO/60 min	NSCs significantly migrate from the core of the lesion to the border of the infarct and stimulate angiogenesis	[26]

Abbreviations: DAPT, γ‐secretase inhibitor; MCAO, middle cerebral artery occlusion; NSCs, neural stem cells; SD, Sprague Dawley; tMCAO, transient middle cerebral artery occlusion.

#### 3.3.3 Corpus callosum

The needle connected to the 5 μL Hamilton syringe was inserted into the corpus callosum of adult male Wistar rats (coordinates A, 0.0; L, 4.0; V, 3.0 mm, where the bregma was adjusted to the same horizontal plane, and the ventral coordinates were calculated by the dura mater), and 2 μL of CMFDA‐labeled HUCB‐NSCs (2 × 10^5^) was injected at a rate of 0.5 μL/min through a microinfusion pump (Stoelting) mounted on the stereotaxic device^42^ (Table 4).

**Table 4:** Representative experimental studies of intracerebral (corpus callosum, cerebral cortex, and hippocampus) NSC transplantation in cerebral ischemia.

Route	Timing of transplantation	Cell type	Dose	Species	Model/Duration	Major finding	Reference
Intracerebral							
Corpus callosum	3 days	Human umbilical cord blood‐derived neural‐like stem cell line (HUCB‐NSC)	2 × 10^5^	Wistar rat	Rat Model of Lacunar Stroke	The occurrence of SVZ and SGZ neurogenesis in the rat brain prolongs the increase in the expression of endogenous nutritional factors induced by focal stroke in the rat brain	[42]
Cerebral cortex	6 h	Mouse NSCs (from the bilateral SVZ)	4 × 10^5^	SD rat	tMCAO/90 min	Minocycline pretreatment improves the survival rate of transplanted cells, promotes the expression of paracrine factors in vitro, and enhances neuroprotection through upregulation of Nrf2	[43]
	7 days	Human NSC series (NSI‐566RSC)	0.5–2 × 10^4^	SD rat	tMCAO/60 min	NSI‐566RSC cell (a spinal cord‐derived NSC line) graft promotes behavioral recovery in stroke animals	[44]
	7 days	Human NSCs (from the telencephalon VZ/SVZ)	1 × 10^5^	C57BL/6J Mouse	dMCAO	The transplanted NSCs directly contributed to the functional recovery of 3K3A‐APC treatment. The combined treatment of advanced NSC and 3K3A‐APC has significant potential for repair after ischemia	[45]
Hippocampus	0 h	Mouse NSCs (from the embryo）	4 × 10^5^	SD rat	MCAO	Promoting synapse remodeling in the brain tissue, improving the functional outcome of stroke mice	[46]
	24 h	Human iPSC‐derived neural progenitor cells (SCC035)	1 × 10^5^	C57BL/6J Mouse	tMCAO/60 min	Quickly migrating to the site of stroke injury, improving neurological dysfunction	[47]

Abbreviations: dMCAO, permanent middle cerebral artery occlusion; MCAO, middle cerebral artery occlusion; NSCs, neural stem cells; SD, Sprague Dawley; SGZ, subgranular zone; SVZ, subventricular zone; tMCAO, transient middle cerebral artery occlusion; VZ, ventricular germinal zone.

#### 3.3.4 Cerebral cortex

The 4 × 10^5^ NSCs were transplanted using a 10 μL Hamilton syringe along the anterior–posterior axis into the cortex at these coordinates: (1) AP, +1.0; ML, +3.0; DV, −3.0; (2) AP, −1.0; ML, +3.0; DV, −3.0; (3) AP, −3.0; ML, +3.0; DV, −2.5; (4) AP, −5.0; ML, +3.0; DV, −2.5.^43^ The coordinates of the cerebral cortex graft site were 0.5 mm anterior to the bregma, 3.5 mm to the right of the suture of the skull, and 3.5 mm below the plane of the skull. 5 × 10^5^ (6 μL) NSCs were implanted in 8–10‐week‐old male mice.^41^ 3 × 10^6^ cells/mL of undifferentiated NSCs were injected on the left side of the skull of adult female Wistar rats with a 10 μL Hamilton syringe through the bregma‐derived cerebral cortex: anterior, 1.0 mm; lateral, 3.0 mm; and ventral, 3.0 mm.^48^ 3 × 10^5^ NSCs were injected into the right cerebral cortex of adult SD rats. Injection target: the coronal suture was used as the center, and the periprosthetic membrane was peeled about 1.5 mm^49^ (Table 4).

#### 3.3.5 Hippocampus

4 × 10^5^ NSCs were injected into the hippocampus around the ischemic border zone of 6–8‐week‐old adult male SD rats at these coordinates: AP, 19 mm; ML, 23.6 mm; and DV, 14.9 mm.^46^ A small burr hole (0.5 mm diameter, F.S.T.) was drilled in the skull of male C57BL/6J mice (2 mm posterior to the bregma, 1.5 mm lateral to the sagittal suture). Two microliters of PBS containing 1 × 10^5^ viable cells were injected over a 3‐min period into the ipsilesional hemisphere (a depth of 2–2.5 mm dorsal)^47^ (Table 4).

#### 3.3.6 Infarct area

5 × 10^4^ NSCs were transplanted into the cortical penumbra of adult male SD rats around the infarct at 1.5 mm posterior to the bregma, 3.0 mm lateral to the midline, and 2.0 mm beneath the dura using a Kopf stereotaxic frame. The transplantation was carried out using a microsyringe to deposit NSCs at a rate of 0.5 mL/min.^11^ 5.0 μL of suspended cells (1 × 10^4^ cells per mL) was injected in the cortical infarct area in adult male SD rats with the Kopf stereotactic frame at 1.5 mm behind the bregma, 3.0 mm outside the midline, and 2.0 mm subdural.^50^ Lentiviral‐transduced 1 × 10^5^ human fetal NSCs were engrafted along the cortical infarct border.^45^

## 4 ROLE OF DIFFERENT INJECTION ROUTES AND DOSES OF STEM CELLS

### 4.1 Cell doses and delivery time

Cell dose is one of the most important variables. According to a review of trials in the literature from Tables 1, 2, 3, the dose of cells in different research varied, generally ranging from 1 × 10^3^ to 1 × 10^6^ cells.^51^ For example, intravenous administration is ranging from 1 × 10^3^ to 1 × 10^6^ cells. Arterial administration is ranging from 1 × 10^5^ to 1 × 10^6^ cells.^20^, ^24^, ^26^, ^28^, ^29^, ^52^ Transplantation of NSCs 3 days after stroke promotes improvements in brain histology and is considered an effective approach to optimize neurological function. NSCs successfully reduced infarct size enlargement and provided optimal neurological outcomes.^51^ At 7 days post‐transplantation onward, there was a significant recovery of motor and neurological functions in stroke animals that received intracerebral transplantation of high doses of 1 × 10^4^ cells/µL and 2 × 10^6^ cells/µL of NSI‐566RSC (a human NSC line) compared to vehicle‐infused stroke animals or those stroke animals that received the low dose of 5 × 10^3^ cells/μL.^44^

It has been shown that the optimal time for stem cell therapy is 3 days after ischemic stroke and that hematopoietic stem cell transplantation may reduce the size of the injury by preventing apoptosis. In addition, NSC transplantation can inhibit infarct size expansion and preserve neurological function.^51^ However, since NSCs therapy for cerebral ischemia is affected by multiple factors, the delivery time of NSC transplantation may be variable in different circumstances.

### 4.2 Comparison of different injection routes

There is no optimal injection route for NSC transplantation. The problem with the delivery of stem cells throughout the body is that only the number of injected cells located at the injury site is very limited. Direct injection is invasive, and although it is a precise method of cell delivery and implantation, it can lead to poor distribution of cells in the target lesion.^53^ Intravenously transplanted human NSCs can reach adult rat brains with ischemic damage and improve functional recovery and long‐term safety.^54^ After intravenous injection, cells are distributed throughout the body, including the liver, spleen, kidney, and spinal cord. This leads to a decrease in the number of cells being delivered to the brain. Carotid artery injection can just circumvent this.^17^ The advantage of arterial injection is that it utilizes the first‐pass effect in the central nervous system to maximize the potential of exogenous nerve cells to settle in the brain, and the surrounding cells first pass through the abundant microcirculation of filter organs such as the lung and liver.^23^ But there are also potential complications: intra‐arterial injection may cause volume overload, which can lead to acute cardiac overload and pulmonary edema. When injected quickly, these risks are magnified and cause damage to the blood vessel wall.^55^ Therefore, both total capacity and speed should be treated with caution, further blocking or interfering with capillaries. Another complication is the formation of NSC embolism in the cerebrovascular system.^23^ Injecting cells directly into the ventricle requires craniotomy with an electric drill, which can cause serious trauma. Compared with intraventricular injection and intravenous injection, intraneck injection shows special advantages, directly promoting cell‐to‐cell contact and inducing TNT formation, and the risk of death is lower than that of intraventricular injection in animal models. In addition, stereotactic intracranial transplantation is to inject NSCs directly into the damaged area. The effect of this route is better than the above methods. However, this approach is invasive and involves a series of operations such as craniotomy, so it may damage nearby brain tissues.^56^ Therefore, there is an urgent need to find a noninvasive method of drug delivery that can effectively penetrate the brain. Although intranasal drug delivery for central system disorders has existed for a long time, intranasal drug delivery using stem cells has only been investigated in recent years. Danielyan et al. in 2009 found that intranasal delivery of stem cells can penetrate the brain of mice and bypass the blood–brain barrier (BBB), a noninvasive route.^57^ Meanwhile, there is growing evidence that stem cells can be reliably delivered to the CNS by intranasal administration, bypassing BBB.^58^, ^59^, ^60^, ^61^, ^62^ In animal models of cerebral ischemia, the repair potential of intranasally administered mesenchymal stem cells has been widely reported.^63^, ^64^, ^65^ For example, human umbilical cord‐derived mesenchymal stem cells (hUC‐MSCs) promote the repair of hypoxia/ischemia‐induced neonatal brain injury in rats.^64^ However, the use of intranasal NSCs for the treatment of cerebral ischemia is still being explored. Fortunately, Gang Ji et al.^66^ found that intranasal transplantation of human NSCs improved neurological function in neonatal rats with hypoxic‐ischemic encephalopathy. Therefore, intranasal administration of NSCs may be a promising noninvasive approach for the treatment of cerebral ischemia.

### 4.3 Importance of NSCs transplantation

After NSCs transplantation, the body has been ameliorated in many aspects. We can obtain a significantly lower Neurological Severity Score (NSS) based on the neurological severity score of NSCs after transplantation, which proves that transplantation of NSC reduces cerebral ischemia and enhances neural function. Furthermore, it improves the migration of NSCs and has a beneficial effect on cerebral ischemia. Meanwhile, nerve function is enhanced after transplantation.^19^, ^22^, ^33^, ^38^, ^51^, ^67^, ^68^, ^69^ Guzman R. et al. tested rotarod performance on CD49 cells. On the 17th day after stroke, in contrast with animals injected with CD49d‐negative cells or vehicle, animals injected with CD49d‐enriched cells demonstrated an obviously better sensorimotor recovery.^17^ NSC transplantation restored the brain function of the stroke mice, so the behavioral test was also significantly improved, and the brain tissue repair was mediated.^25^ In addition, NSCs migrate into the ischemic brain maintaining proliferative ability, which can maintain a strong proliferation ability in an ischemic environment for at least 14 days. NSCs also suppress ischemia‐triggered inflammatory cytokines and reduce the volume of cerebral infarction.^46^ NSC transplantation stimulated neurogenesis and endogenous angiogenesis in aged ischemic rats.^31^ At the same time, it can promote the angiogenesis of the cortical infarction area, and make NSC differentiate into neurons, astrocytes, and oligodendrocytes in vivo, so as to participate in the repair of nerve tissues.^19^, ^23^, ^50^, ^67^ In addition, they can differentiate into non‐nerve cells by transgerminal differentiation.^70^ Animals transplanted with fetal NSCs displayed significantly better performance not only in limb placement tests and functional scores, but also in differentiation into axons, dendrites, and astrocytes beneath both in vitro and vivo conditions.^24^

### 4.4 NSCs transplantation combined with adjuvant therapeutic methods

There are many methods that can be used to promote the effect of NSC transplantation. Some methods are as follows: Neurological severity score was performed after BDNF‐NSCs transplantation, which improved neurological function to a greater extent.^27^ The silencing of circular RNA HIPK2 (CircHIPK2) expression can enhance functional recovery and NSCs differentiation into neurons after stroke, as well as promote neuronal plasticity.^29^, ^67^ Meanwhile, AF‐NSCs were transplanted into ischemia rats and induced functional recovery. miR‐145 protects the function of neuronal stem cells and enhances the activity of NSCs by targeting the mitogen‐activated protein kinase (MAPK) pathway to treat cerebral ischemic stroke in rats. Overexpression of miR‐145 promotes the proliferation and differentiation of NSCs while inhibiting cell apoptosis.^49^ After iPSC‐derived NSCs are stereotactically implanted into the brain, the implanted cells can survive, migrate to the ischemic brain area, and differentiate into mature nerve cells.^34^ After acupuncture + NSCs transplantation, the apoptosis of nerve cells decreases and the survival rate of NSCs increases.^31^, ^33^, ^71^ CoMo‐NSCs efficiently self‐renew and generate neurons and glia in vitro.^72^ DAPT can promote the differentiation of transplanted NSCs into neurons and has a neuroprotective effect on NSCs transplantation in rats with cerebral ischemia.^73^ Transplantation of PCNSCs enhanced neuroprotection in ischemic stroke.^44^ At last, pretreatment will further enhance the effect of NSCs after NSCs transplantation.^56^

## 5 CONCLUSIONS

This review focuses on the various methods, doses, timing, and effects of NSC injections in cerebral ischemia. The optimal injection site, timing, and dose for NSCs transplantation after cerebral ischemia remain controversial as the effect of NSCs injection is influenced by several factors. In terms of injection routes, both intravenous and arterial injections are noninvasive. Intracerebral and stereotactic injections are invasive and can cause severe trauma, especially when administered intracerebrally. Intracerebral stereotactic injections are more effective than other methods but carry greater clinical risk. Although intranasal administration is a noninvasive method of delivery, there are fewer studies of intranasal administration of NSCs for the treatment of cerebral ischemia, which may be a promising alternative cell therapy for the treatment of cerebral ischemia. In terms of cell dose, different studies have different cell doses, typically 1 × 10^3^–1 × 10^6^ cells. In terms of timing of injection, the optimal time for stem cell therapy is about 3 days after ischemic stroke. However, the optimal time of injection may vary due to various factors. In addition, cotransplantation and promotion of stem cell derivatives are strategies to improve the survival rate of NSCs transplanted. Taken together, these routes of administration to deliver stem cells and/or therapeutic substances to the damaged site need to be further optimized for better clinical application.

## AUTHOR CONTRIBUTIONS

Xing‐Yu Yang, Li Chen, and Jun‐Feng Cao conceived and designed the work leading to the submission. Sheng‐Yan Chen, Mei Wu, Jun‐Feng Cao, and Xing‐Yu Yang checked the information and summarized the needs of the authors. Xing‐Yu Yang, Jun‐Feng Cao, and Mei Wu made a summary diagram. Xing‐Yu Yang, Sheng‐Yan Chen, and Mei Wu made the table. Xing‐Yu Yang drafted the manuscript. Xing‐Yu Yang, Sheng‐Yan Chen, Mei Wu, and Li Chen revised the manuscript. Li Chen and Xiao Zhang approved the final version.

## CONFLICT OF INTEREST STATEMENT

The authors declare no conflict of interest.

## ETHICS STATEMENT

Not applicable.

## TRANSPARENCY STATEMENT

All the authors affirm that this manuscript is an honest, accurate, and transparent account of the study being reported.

## Data Availability

Data sharing is not applicable to this review as no new data were created in this study.
